# Supplementary material for: The Small RNA Universe of Capitella teleta
Source: Front Mol Biosci. 2022 Feb 25;9:802814. doi: 10.3389/fmolb.2022.802814 (PMC8915122; doi:10.3389/fmolb.2022.802814)
Supplement: Supplementary file 1 [file DataSheet1.ZIP › Supplement/known/CAPTEscaffold_12_1214.pdf]

Provisional ID : CAPTEscaffold_12_1214
Score total : 27549.3
Score for star read(s) : 3.9
Score for read counts : 27539.4
Score for mfe : 1.4
Score for randfold : 1.6
Score for cons. seed : 3
Total read count : 54029
Mature read count : 53798
Loop read count : 0
Star read count : 231

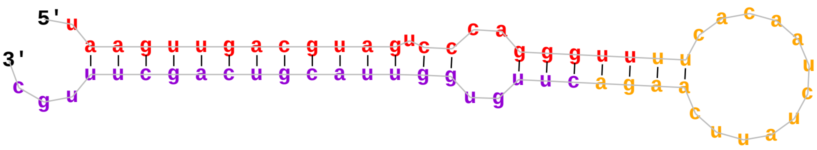

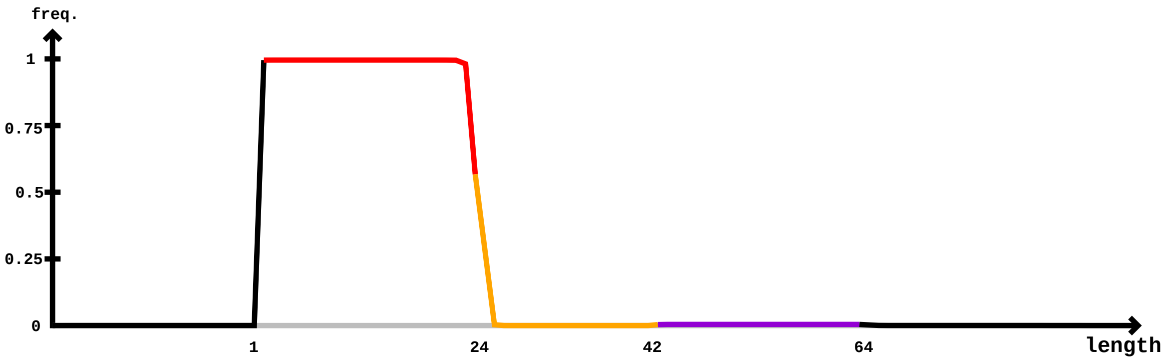


```
                     Mature                          Star
5'- uccucugcucacacagugucuaaguugacguagucccaggguuuucacaaucuauucaagacuugugguuacgucagcuuugccauuguugggcacugcuucuuccccugc  -3'  obs
    uccucugcucacacagugucuaaguugacguagucccaggguuuucacaaucuauucaagacuugugguuacgucagcuuugccauuguugggcacugcuucuuccccugc       exp
    uccucugcucacacagugucuaaguugacguagucccaggguuuucacaaucuauucaagacuugugguuacgucagcuuugccauuguugggcacugcuucuuccccugc       known
    .....((((((.((((((.(.((((((((((((.((..(((((((...........)))))))..))))))))))))))).).))))))))))))...............   reads   mm   sample

    uccucugcucacacaguguc...........................................................................................   4       0    seq
    ..................uGuaaguugacguagucccagg.......................................................................   1       1    seq
    ..................ucuaaguugacguagucccagggu.....................................................................   2       0    seq
    ....................uaaguugacguagucccag........................................................................   2       0    seq
    ....................uaaguugacguagucccagU.......................................................................   1       1    seq
    ....................uaaguugacguagucccagA.......................................................................   2       1    seq
    ....................uaaguugacguagucccagg.......................................................................   25      0    seq
    ....................uaaguugacguaguUccaggg......................................................................   1       1    seq
    ....................uaaguugacguagucAcaggg......................................................................   2       1    seq
    ....................uaaguugacguagucccaggg......................................................................   727     0    seq
    ....................uaaguugacguagucccagAg......................................................................   2       1    seq
    ....................uaaguugacguagucccaAgg......................................................................   1       1    seq
    ....................Naaguugacguagucccaggg......................................................................   1       1    seq
    ....................Aaaguugacguagucccaggg......................................................................   2       1    seq
    ....................uaaguugacguagucccaggA......................................................................   7       1    seq
    ....................uaaguugacguGgucccaggg......................................................................   1       1    seq
    ....................uaaguugacguagucccaggU......................................................................   1       1    seq
    ....................uaaguugaUguagucccaggg......................................................................   1       1    seq
    ....................uaaguugGcguagucccagggu.....................................................................   1       1    seq
    ....................uaaguugacguagucccagggu.....................................................................   21573   0    seq
    ....................uaaguugacguGgucccagggu.....................................................................   5       1    seq
    ....................uaaguugacguagucccaAggu.....................................................................   13      1    seq
    ....................uaaguugUcguagucccagggu.....................................................................   3       1    seq
    ....................uaagCugacguagucccagggu.....................................................................   3       1    seq
    ....................uaaguugacguagAcccagggu.....................................................................   1       1    seq
    ....................Gaaguugacguagucccagggu.....................................................................   12      1    seq
    ....................uaaguugacguaguccUagggu.....................................................................   18      1    seq
    ....................uaaguugacgAagucccagggu.....................................................................   1       1    seq
    ....................uaaguugacguagCcccagggu.....................................................................   1       1    seq
    ....................uaaguugacguaAucccagggu.....................................................................   3       1    seq
    ....................uaaguugacguagucccaggAu.....................................................................   1       1    seq
```

```
                    Mature                                    Star
uccucugcucacacagugucuaaguugacguagucccaggguuuucacaaucuauucaagacuugugguuacgucagcuuugccauuguugggcacugcuucuuccccugc
....................uaGguugacguagucccagggu.....................................................................	3	1	seq
....................uaaguugacguaguGccagggu.....................................................................	2	1	seq
....................uNaguugacguagucccagggu.....................................................................	4	1	seq
....................Naaguugacguagucccagggu.....................................................................	3	1	seq
....................uaaguugacguaguAccagggu.....................................................................	5	1	seq
....................uaaguugacguagucUcagggu.....................................................................	19	1	seq
....................uaaguugacguagucccaggUu.....................................................................	3	1	seq
....................uaaguugacguagucGcagggu.....................................................................	3	1	seq
....................uaaguugacguCgucccagggu.....................................................................	101	1	seq
....................uaaguugacgCagucccagggu.....................................................................	1	1	seq
....................uaaguugacguagucccaCggu.....................................................................	2	1	seq
....................uaaguuCacguagucccagggu.....................................................................	1	1	seq
....................uaaguAgacguagucccagggu.....................................................................	7	1	seq
....................uaaguugacguagucccagggA.....................................................................	288	1	seq
....................uaaguugacguagucAcagggu.....................................................................	10	1	seq
....................uaaguugaAguagucccagggu.....................................................................	3	1	seq
....................uaaguugacguaguUccagggu.....................................................................	2	1	seq
....................uGaguugacguagucccagggu.....................................................................	2	1	seq
....................uaaguugacguagucccagggG.....................................................................	59	1	seq
....................uaaAuugacguagucccagggu.....................................................................	2	1	seq
....................uaaguugacguagucccagCgu.....................................................................	1	1	seq
....................uaaguugacguaguccAagggu.....................................................................	7	1	seq
....................Aaaguugacguagucccagggu.....................................................................	102	1	seq
....................uaaguNgacguagucccagggu.....................................................................	1	1	seq
....................uaaguugacAuagucccagggu.....................................................................	7	1	seq
....................Caaguugacguagucccagggu.....................................................................	4	1	seq
....................uaaguugacguagucccUgggu.....................................................................	2	1	seq
....................uaaguugacguagucccagggC.....................................................................	2	1	seq
....................uaaguugaUguagucccagggu.....................................................................	4	1	seq
....................uaaguuAacguagucccagggu.....................................................................	4	1	seq
....................uaaguugacguaguccGagggu.....................................................................	2	1	seq
....................uaaguugacguagucccagAgu.....................................................................	15	1	seq
....................uaaguugaGguagucccagggu.....................................................................	5	1	seq
....................uaaguCgacguagucccagggu.....................................................................	5	1	seq
....................uaaguugCcguagucccagggu.....................................................................	4	1	seq
....................uaaguugacguaUucccagggu.....................................................................	2	1	seq
....................uaaguugacCuagucccagggu.....................................................................	3	1	seq
....................uaaguugacguagucccGgggu.....................................................................	3	1	seq
....................uaaguugacguagucccaggCu.....................................................................	3	1	seq
....................uaUguugacguagucccagggu.....................................................................	1	1	seq
....................uaagAugacguagucccagggu.....................................................................	16	1	seq
....................uaaguugacguagucccaUggu.....................................................................	2	1	seq
....................uaaguugacguagucccagUguu....................................................................	3	1	seq
....................uaaguugaGguagucccaggguu....................................................................	7	1	seq
....................Naaguugacguagucccaggguu....................................................................	12	1	seq
....................uaaguugacUuagucccaggguu....................................................................	2	1	seq
....................uaaguugacguagucccaggguG....................................................................	6	1	seq
....................uaaguugaUguagucccaggguu....................................................................	15	1	seq
....................uaaguugacgCagucccaggguu....................................................................	5	1	seq
....................Caaguugacguagucccaggguu....................................................................	4	1	seq
....................uaaguugacguagucccaggAuu....................................................................	2	1	seq
....................uaaAuugacguagucccaggguu....................................................................	2	1	seq
....................uaaguNgacguagucccaggguu....................................................................	1	1	seq
....................uaaguugacguagucccagCguu....................................................................	3	1	seq
....................uaaguugacguaguGccaggguu....................................................................	1	1	seq
....................uaaguuAacguagucccaggguu....................................................................	5	1	seq
....................uaaguugacguaguccUaggguu....................................................................	21	1	seq
....................uaaguugacguaguUccaggguu....................................................................	4	1	seq
....................uaaguugacguagCcccaggguu....................................................................	3	1	seq
....................uaaguugacguGgucccaggguu....................................................................	6	1	seq
....................uaaguugUcguagucccaggguu....................................................................	1	1	seq
....................uaaguugacguagucccagAguu....................................................................	12	1	seq
....................uaaguugacguaguccAaggguu....................................................................	7	1	seq
....................uaaguuCacguagucccaggguu....................................................................	2	1	seq
....................uaaguugacguagAcccaggguu....................................................................	3	1	seq
....................uaaguugaAguagucccaggguu....................................................................	1	1	seq
....................uaaguugacguaUucccaggguu....................................................................	1	1	seq
....................uaaguCgacguagucccaggguu....................................................................	31	1	seq
....................uaaguugGcguagucccaggguu....................................................................	5	1	seq
....................uaaguugacguagucccaggguA....................................................................	1409	1	seq
```

Mature **Star**

```
uccucugcucacacagugucuaaguugacguagucccaggguuuucacaaucuauucaagacuugugguuacgucagcuuugccauuguugggcacugcuucuuccccugc
....................uaagAugacguagucccaggguu....................................................................  16     1  seq
....................uaagGugacguagucccaggguu....................................................................  1      1  seq
....................uaaguugacguagucccaggguu....................................................................  25888  0  seq
....................Aaaguugacguagucccaggguu....................................................................  132    1  seq
....................uNaguugacguagucccaggguu....................................................................  1      1  seq
....................uaaguugacguagucccaAgguu....................................................................  13     1  seq
....................uaaguugacguaguccGaggguu....................................................................  1      1  seq
....................Gaaguugacguagucccaggguu....................................................................  12     1  seq
....................uaagCugacguagucccaggguu....................................................................  2      1  seq
....................uGaguugacguagucccaggguu....................................................................  6      1  seq
....................uaaguugacguagucccagggGu....................................................................  7      1  seq
....................uaaguugCcguagucccaggguu....................................................................  2      1  seq
....................uaaguugacguagucccUggguu....................................................................  3      1  seq
....................uaaguugacgAagucccaggguu....................................................................  6      1  seq
....................uaaguugacguagucccagggCu....................................................................  2      1  seq
....................uaaguugacCuagucccaggguu....................................................................  2      1  seq
....................uaaguugacguagucAcaggguu....................................................................  8      1  seq
....................uaaguugacguagucccaggguC....................................................................  8      1  seq
....................uaaguugacguaAucccaggguu....................................................................  3      1  seq
....................uaaguugacguaCucccaggguu....................................................................  2      1  seq
....................uaaguugacAuagucccaggguu....................................................................  1      1  seq
....................uaaguAgacguagucccaggguu....................................................................  6      1  seq
....................uaaguugacguagucGcaggguu....................................................................  4      1  seq
....................uaaguugacguagucccaggCuu....................................................................  1      1  seq
....................uaaguugacguagucccagggAu....................................................................  8      1  seq
....................uaaguugacguagucccGggguu....................................................................  3      1  seq
....................uaaguugacguagucccagNguu....................................................................  1      1  seq
....................uaaguugacguagucUcaggguu....................................................................  24     1  seq
....................uaaguugacguagucccaggCuuu...................................................................  1      1  seq
....................uaaguugacguagucccaggguAu...................................................................  9      1  seq
....................uaaguugaUguagucccaggguuu...................................................................  1      1  seq
....................uGaguugacguagucccaggguuu...................................................................  1      1  seq
....................uaaCuugacguagucccaggguuu...................................................................  1      1  seq
....................uaaguugacguaguccUaggguuu...................................................................  3      1  seq
....................uaaguugGcguagucccaggguuu...................................................................  2      1  seq
....................uaaguugacguagucccaggguuA...................................................................  1563   1  seq
....................uaaguugacguaguccAaggguuu...................................................................  1      1  seq
....................uaaguugacguagucccaggguuu...................................................................  1157   0  seq
....................uaaguugacguagucccaggguuG...................................................................  1      1  seq
....................uaaguugacguagucUcaggguuu...................................................................  1      1  seq
....................uaaguugaAguagucccaggguuu...................................................................  1      1  seq
....................uaaguugacguagucAcaggguuu...................................................................  1      1  seq
....................Aaaguugacguagucccaggguuu...................................................................  2      1  seq
....................uaaguugacguagucccaUgguuu...................................................................  1      1  seq
....................uaagAugacguagucccaggguuu...................................................................  1      1  seq
....................uaaguugacguagucccagggAuu...................................................................  1      1  seq
....................uaaguugacguagucccaggguuAu..................................................................  121    1  seq
....................uaaguugacguagucccaggguuCu..................................................................  3      1  seq
....................uaaguugacguagucccaggguAuu..................................................................  2      1  seq
....................uaaguugacguagucccaggguuuA..................................................................  8      1  seq
....................uaaguugacguagucccaggguuuu..................................................................  35     0  seq
....................uaaguugacguagucccaggguuuuU.................................................................  4      1  seq
....................uaaguugacguagucccaggguuuucU................................................................  2      1  seq
.....................aaguugacguagucccagggu.....................................................................  4      0  seq
.....................Uaguugacguagucccagggu.....................................................................  2      1  seq
.....................aaguugacguagucccaggguu....................................................................  2      0  seq
........................uugacguagucccagggu.....................................................................  1      0  seq
.............................................................cuugugguuacgucagcuu...............................  1      0  seq
.............................................................cuugugguuacgucagcuuug.............................  5      0  seq
.............................................................cuugugguuacgucagcuuuUc............................  1      1  seq
.............................................................cAugugguuacgucagcuuugc............................  1      1  seq
.............................................................cuugugguuacgucagcuuugc............................  181    0  seq
.............................................................cuugugguuacgucagcuuugcA...........................  1      1  seq
.............................................................cuugugguuacgucagcuuugcU...........................  2      1  seq
..............................................................uugugguuacgucagcuuugc............................  1      0  seq
..............................................................uugugguuacgucagcuuugcc...........................  18     0  seq
..............................................................uuAugguuacgucagcuuugcc...........................  1      1  seq
..............................................................uugugguuacgucagcuuugccU..........................  19     1  seq
...................................................................................cauuguugggcacugcuuc.........  1      0  seq
...................................................................................cauuguugggcacugcuucu........  4      0  seq
```

```
                    Mature                                  Star
uccucugcucacacagugucuaaguugacguagucccagguuuucacaaucuauucaagacuugugguuacgucagcuuugccauuguugggcacugcuucuuccccugc
..................................................................................cauuguugggcacugcuucuucc.....    2    0    seq
..................................................................................cauuguugggcacugcuucuuccccugc    2    0    seq
```
